# diffMONT: predicting methylation-specific PCR biomarkers based on nanopore sequencing data for clinical application

**DOI:** 10.1093/bioinformatics/btag039

**Published:** 2026-01-22

**Authors:** Daria Meyer, Emanuel Barth, Laura Wiehle, Manja Marz

**Affiliations:** RNA Bioinformatics and High-Throughput Analysis, Friedrich Schiller University Jena, Jena 07743, Germany; Bioinformatics Core Facility, Friedrich Schiller University Jena, Jena 07743, Germany; Oncgnostics GmbH, Jena 07749, Germany; RNA Bioinformatics and High-Throughput Analysis, Friedrich Schiller University Jena, Jena 07743, Germany; Bioinformatics Core Facility, Friedrich Schiller University Jena, Jena 07743, Germany; Oncgnostics GmbH, Jena 07749, Germany; RNA Bioinformatics and High-Throughput Analysis, Friedrich Schiller University Jena, Jena 07743, Germany; Bioinformatics Core Facility, Friedrich Schiller University Jena, Jena 07743, Germany; Fritz Lipmann Institute, Leibniz Institute on Aging, Jena 07745, Germany; European Virus Bioinformatics Center, Jena 07743, Germany; Balance of the Microverse, Cluster of Excellence, Jena 07745, Germany

## Abstract

**Motivation:**

DNA methylation serves as a key biomarker in clinical diagnostics, especially in cancer detection. With methylation-specific PCR (MSP), a widely used approach, patient samples can be screened fast and efficiently for differential methylation. During MSP, methylated regions are selectively amplified with specific primers. With nanopore sequencing, knowledge about DNA methylation is generated during direct DNA sequencing without needing pretreatment of the DNA. Multiple methods, mainly developed for whole-genome bisulfite sequencing (WGBS) data, exist to predict differentially methylated regions (DMRs) in the genome. However, the predicted DMRs are often very large and not sufficiently discriminating to generate meaningful results in MSP, creating a gap between theoretical cancer marker research and practical application, as no tool currently provides methylation difference predictions tailored for PCR-based diagnostics.

**Results:**

Here, we present **diffMONT**, a tool that predicts differentially methylated regions specifically suited for MSP primer design, enabling rapid translation into practical applications. **diffMONT** takes into account (i) the specific length of primer and amplicon regions, (ii) the fact that one condition should be unmethylated, and (iii) a minimal required amount of differentially methylated cytosines within the primer regions. We compared the results of **diffMONT** to **metilene** and **DSS** based on a publicly available nanopore sequencing dataset and show that the regions predicted by **diffMONT** are more specific toward hypermethylated regions. **diffMONT** accelerates the design of methylation-specific diagnostic assays, bridging the gap between theoretical research and clinical application.

**Availability and implementation:**

The source code for **diffMONT**, an open-source Python-based tool, is available at https://github.com/rnajena/diffMONT/, with an archived release under https://zenodo.org/records/17641031.

## 1 Introduction

In cancer research, the accurate detection of DNA methylation is crucial for understanding tumorigenesis and for developing diagnostic and therapeutic strategies. One approach used in clinical diagnostic tests is methylation-specific PCR (MSP) (Herman *et al.* 1996), which uses bisulfite treatment, converting unmethylated cytosines to uracils while leaving methylated cytosines unchanged, allowing for the differentiation between methylated and unmethylated DNA by specific primers only binding to methylated sites. MSP is both simple and economical, making it suitable for population-wide screening (Locke *et al.* 2019). The design of MSP primers has to balance (i) specificity, to avoid creating unspecific PCR products or false positives in healthy individuals, and (ii) efficiency, to achieve the theoretical maximum of duplication in each amplification step.

**Figure 1:**
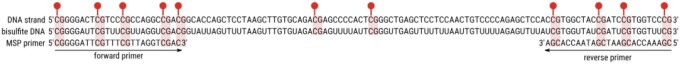
Standard primer design workflow, assuming all CpG dinucleotides are completely methylated. By treating the DNA with sodium-bisulfite (bisulfite), all non-methylated cytosines are deaminated into uracils. In a following PCR, the methylation specific primers bind only to methylated DNA stretches. Thus, only methylated DNA stretches are amplified, unmethylated DNA is not amplified. This allows a methylation-specific amplification of DNA. Primer length: 25 nt, PCR product length: 120 nt.

For MSP in clinical contexts, a primer length of 20 to 30 nt is used (Dieffenbach *et al.* 1993, Li 2007), with resulting PCR products of 120 to 400 bp length (Dieffenbach *et al.* 1993, Li 2007). The GC content of the PCR primers should be moderate, with 50%–60% being ideal (Singh and Kumar 2001). For bisulfite-conversion-based PCR the primer sequence should also have enough non-CpG cytosines (Herman *et al.* 1996). Figure 1 shows an example of an MSP primer pair design for a given DNA sequence.

For the detection of marker regions for MSP, ONT sequencing is advantageous as it considers the entire genome, providing a comprehensive, unprejudiced view beyond just the regions described in literature or known disease-associated genes. This method surpasses the limitations of focusing solely on known regions, offering a broader and more inclusive analysis, while also directly measuring DNA methylation without the need of an amplification or conversion step on a single nucleotide resolution (Simpson *et al.* 2017). In contrast, whole genome bisulfite sequencing (WGBS) can introduce a bias by preferentially amplifying DNA with low GC-content (Aird *et al.* 2011) and under-amplifying methylated DNA (Kiselev *et al.* 2015), resulting in an uneven representation of DNA fragments. While the cost and sensitivity of nanopore sequencing currently limit its diagnostic use, it is well-suited for detecting differentially methylated regions using tools developed for short or long-read sequencing (Jühling *et al.* 2016, Park and Wu 2016, Akbari *et al.* 2021, Snajder *et al.* 2023). These regions could potentially serve as targets for MSP.

A variety of bioinformatic tools are available for measuring DNA methylation differences, primarily designed for short reads or bisulfite data analysis, like methylKit (Akalin *et al.* 2012), DSS (Feng *et al.* 2014), metilene (Jühling *et al.* 2016), methCP (Gong and Purdom 2020), DMRcate (Peters *et al.* 2021), HMST-Seq-Analyzer (Farooq *et al.* 2020), or pycoMeth (Snajder *et al.* 2023).

Dispersion Shrinkage for Sequencing data (DSS) (Feng *et al.* 2014) has an integrated beta-binomial regression model with an “arcsine” link function, utilizing a Bayesian hierarchical framework for differential methylation analysis and assesses per-cytosine significance using a Wald test. However, DSS has been noted in a comparative study for its slower computational speed and potential memory issues, as well as a tendency to identify regions that may exceed the actual size of DMRs (Gong and Purdom 2020).

metilene (Jühling *et al.* 2016) is designed for predicting DMRs with high-speed performance and efficient memory usage (Jühling *et al.* 2016, Condon *et al.* 2018) to efficiently process large datasets. It has a binary segmentation algorithm implemented, coupled with a 2D statistical test for detecting DMRs. While metilene is not effective for analyzing methylation differences in single-CpG analysis (Piao *et al.* 2021), it is known to identify DMR boundaries more accurately than DSS (Piao *et al.* 2021) and work well with low coverage data. However, it has been noted to identify numerous DMRs with low mean methylation differences between groups, although this parameter can be adjusted (Gong and Purdom 2020).

The various analysis tools for identifying differential methylation patterns differ significantly in performance, including speed, memory usage, number of sample groups to compare, coverage, and the ability to detect small effect sizes. However, most of these tools are not suitable for the analysis of clinical low coverage nanopore sequencing data, obtained from whole genome sequencing on a standard MinION nanopore flow cell, which results in a sequencing depth below 10×.

To date, no tool has been specifically designed for the adequate detection of primer binding sites or the prediction of potential biomarkers that can be easily and affordably detected using methylation-specific PCR (MSP). Especially, identifying DNA methylation marker regions that can be effectively translated into clinical practice remains a significant challenge (Locke *et al.* 2019). Biomarkers not only need to be clearly differentially methylated in disease but also must be suitable for clinical diagnostic tests, ensuring that healthy individuals are minimally misclassified as having cancer, by minimizing the number of false positive test results and at the same time maximizing detection rates of diseased individuals.

Here, we present the workflow diffMONT, written in Python, which addresses critical gaps in DNA methylation marker discovery and validation, particularly for clinical diagnostic via MSP. diffMONT predicts potential diagnostic biomarkers based on low-coverage nanopore sequenced whole genome DNA methylation data independent of the used methylation caller, considering clinical constraints by minimizing false positives in the prediction of candidate regions and applying it to clinical datasets. diffMONT allows for customization of parameters such as methylation thresholds, region length, minimum differential CpG sites, and PCR product size. Using an Oxford Nanopore Technology (ONT) dataset for validation, our tool was benchmarked against the established DMR calling tools metilene and DSS, demonstrating fewer regions detected, however, with more restricted methylation differences and direct applicability for MSP.

## 2 Materials and methods

### 2.1 The diffMONT pipeline

diffMONT is a Python-based tool designed for analyzing DNA methylation differences between a control group (e.g. healthy individuals) and a disease group (e.g. patients with the same cancer type) and is available for download on GitHub (https://github.com/rnajena/diffONT/). It evaluates methylation variations across the entire genome, taking into account sequencing coverage and specifically identifying regions where methylation is absent in the control group but elevated in the disease group. diffMONT outputs and scores potential biomarker regions independent of their location relative to known genes, including upstream, downstream, intergenic, and gene body regions. Additionally, it suggests primer sequences suitable for methylation-specific PCR (MSP) to facilitate experimental validation of identified potential biomarkers.

#### 2.1.1 Data preprocessing

**Figure 2:**
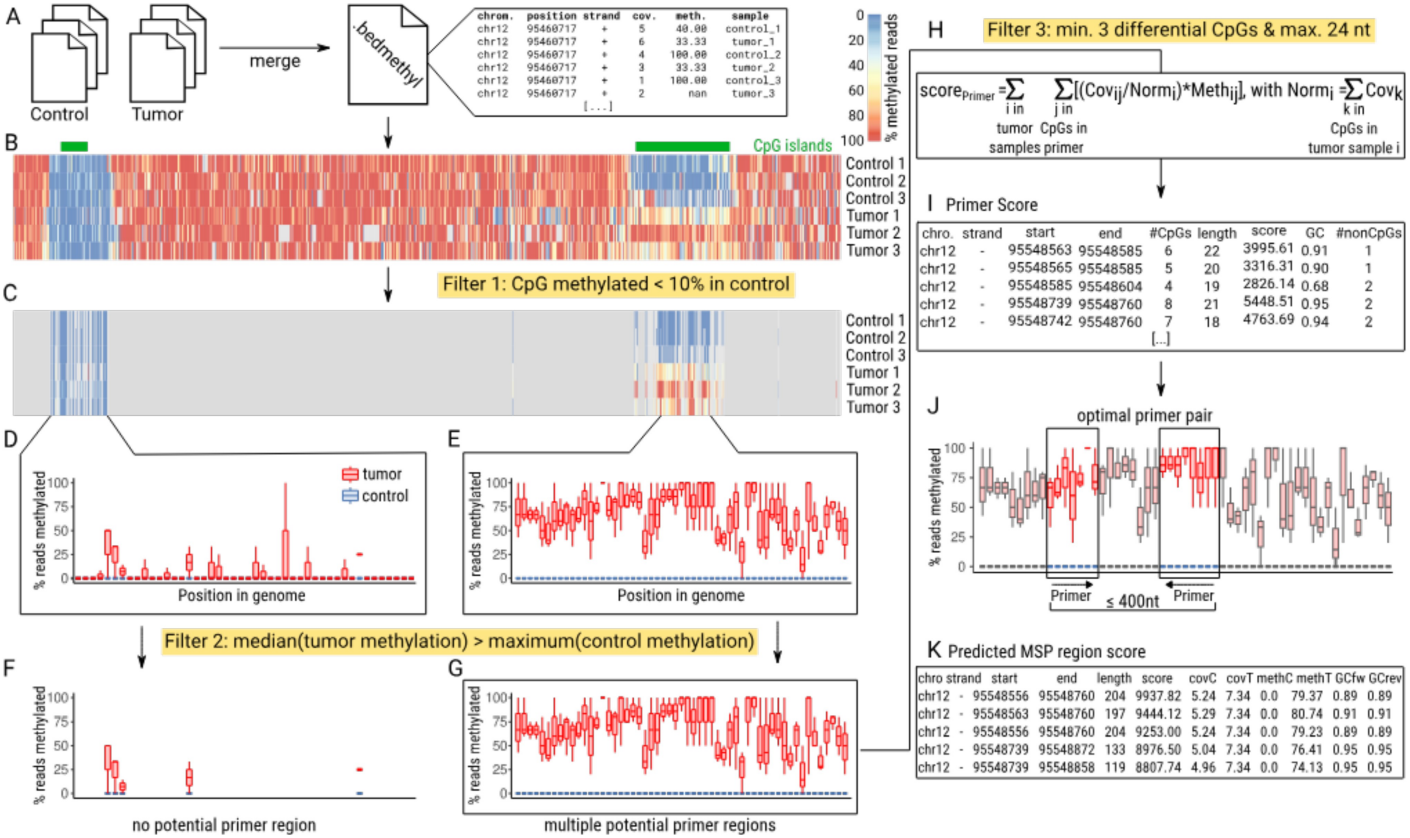
The diffMONT workflow detects potential MSP regions, which differentiate between disease and control samples by containing CpG positions being unmethylated in control and methylated in disease samples (median methylation in disease samples above maximum methylation in control samples). Required input data are one bedmethyl file (e.g. generated by modbam2bed) per sample, containing information about number of reads and percentage methylation per cytosine for one sample. (A) In a preproccessing step bedmethyl files of different samples are merged and sorted by genomic position into one bedmethyl file. (B) Visualization of CpG methylation information for an exemplary region [exclusively (+) strand data are shown]; UCSC CpG islands are annotated in green. (C) The methylation in the control samples is analyzed for each position. If the methylation exceeds the user-defined threshold (default >10%) in only one of the control samples, the position is excluded from further analysis, Filter 1. (D, E) Boxplot statistics (0th, 25th, 50th, 75th, and 100th percentile) are calculated for disease and control samples of each remaining position. These statistics are provided as tables to the user. (F, G) Only positions where the 50th percentile methylation in disease is above the 100th percentile methylation in control are considered as CpGs of interest. These are screened for potential primer regions. Primer regions should contain at least three differentiating CpGs within (per default) at most 24 nt. While for (F) the distance between the remaining CpGs is too large to form primers, for (G) multiple primers are possible. (H) For all potential primer regions a score is calculated, by summing up the normalized coverage multiplied with the percentage methylation for all remaining cytosine positions within the primer for all disease samples. (I) The table of potential primers and their scores is provided to the user. (J) The best combination of primers is selected based on the highest summed score of two primers within a user-defined region (default 60–400 nt). (K) The score for these MSP regions is determined by summing up the scores of the individual primers. The list containing predicted MSP region scores, GC content, coverage, and MSP region length is provided to the user.

diffMONT requires a merged and sorted bedmethyl file as input using the --bedmethyl parameter. A bedmethyl file contains for each sample for each position and for each strand information about coverage and methylation percentage (Fig. 2A and B) and can be generated from both nanopore sequencing data as well as WGBS data. The bedmethyl file should contain data from at least one control and one disease sample with an additional column for the sample name. It can be generated with the script preprocess.py, which merges multiple bedmethyl files into one bedmethyl file, and sorts it by genomic positions. An additional column defines the origin of the entry for each line.

#### 2.1.2 Filter 1: non-methylated CpGs in control samples

For the usage in a clinical context, we aim to reduce the number of false positives and require the control samples to show almost no methylation and diseased samples being hypermethylated. Further, we expect that the larger the difference between the two conditions, the more robust the MSP results will be, when using the predicted primers as biomarkers in a clinical screening context. Therefore, we implemented a filter, which removes from the bedmethyl file all positions with a methylation above 10% (default of --minCpGs) over all reads mapping to this position in at least one control sample, Fig. 2C. This cutoff can be adapted by the user to increase or decrease specificity by setting a lower or higher methylation threshold, respectively, in control samples. Adaption might be preferable if (i) the input data quality changes or (ii) different PCR assays are used, e.g. when using additional TaqMan probing. Additionally, with the parameters --minCtrCov and --minCtrls a minimum coverage (default 3×) for a minimum number of control samples (default 50% of total control samples) can be defined.

#### 2.1.3 Calculation of boxplot data

Next, for all remaining positions, the methylation values for the disease and control samples are aggregated per position and visualized as boxplots (Fig. 2D–G). The 25th and 75th (equals upper and lower quartile, respectively) are calculated with the numpy function nanpercentile, the 50th percentile (equals median) with the function nanmedian. NAN values, where no methylation status could be determined for a sample and position were not taken into account in this way. For the calculation of the 0th and 100th percentile we use the interquartile range *iqr *= 75th percentile—25th percentile. The 0th percentile was calculated as the lowest value above 25th percentile −1.5·iqr. The 100th percentile was calculated as the highest value below 75th percentile +1.5·iqr. The 0th and 100th percentile represent the highest and lowest value, excluding outliers.

#### 2.1.4 Filter 2: selection for differentiating CpGs

We remove all CpGs, which show no strong differences between disease and control sample. Thus, for each position the median disease methylation (50th percentile) has to be above the 100th percentile of the control sample. As a result, most arbitrary genomic regions are removed as they only show moderate elevation of disease methylation levels, see Fig. 2D and F. Potential biomarker regions are expected to be hypermethylated over a longer distance in diseased samples, see Fig. 2E and are subjected to the rest of the workflow, see Fig. 2E and G.

#### 2.1.5 Filter 3: selection of MSP primer suitable regions

Regions for the MSP primer design are selected based on a defined (i) primer length (default 18–24 nt by --minPrimerLength and --maxPrimerLength) and a (ii) minimum number of differentially methylated cytosines (--minCpGs default 3). Thus, the genome is screened for regions which have at least three positions being methylated in the disease samples and unmethylated in the control samples within at most 24 nucleotides. The selected potential MSP regions can contain multiple possible primer positions, see Fig. 2G.

#### 2.1.6 Score calculation

For all potential primer regions, a score is calculated based on the disease sample coverage and disease sample methylation, see Fig. 2H and I. The primer score is calculated as:


$$\mathrm{scor}e_{\mathrm{Primer}}=\sum_{i=1}^{N}\sum_{j=1}^{M}\frac{Cov_{ij}}{\mathrm{Nor}m_{i}}*\mathrm{Met}h_{ij}$$

$$\mathrm{Nor}m_{i}=\sum_{k=1}^{K}Cov_{k}$$

with *N* = number of disease samples, *M* = number of CpGs within the primer, and *K* = number of CpGs within disease sample *i*. For all remaining CpGs within a primer, the normalized coverage is multiplied by the methylation percentage for each disease sample. In this way, primers with highly covered and highly methylated cytosines in the disease samples result in a high score. As the control methylation has already been checked in Filter 1 and 2, only the disease sample methylation is taken into account. The coverage is normalized to be comparable between all disease samples to receive relative coverage values for each disease sample.

#### 2.1.7 Primer pair formation

All potential primer combinations resulting in MSP regions with length between 60 and 400 nt (default, including primers, addressed by --minAmpliconLength and --maxAmpliconLength) are extracted, with the score being the sum of the two corresponding primer scores, see Fig. 2J and K. Overlapping primer pairs are possible and desired due to a combination of different annealing temperatures and other user-specific requirements.

#### 2.1.8 Further output

By default, diffMONT produces four output files: boxplotData.tsv contains aggregated methylation information per position for all differentially methylated cytosines that meet the criteria of Filter 1 and Filter 2. interestingCpGs.tsv provides per sample, per position coverage and methylation information extracted from the bedmethyl file for all differentially methylated cytosines that fulfill the criteria of Filter 1 and Filter 2. primerData.tsv lists information for each predicted MSP primer. pcrProduct.tsv includes details for every predicted MSP region. If the program is run multiple times, these files will be overwritten unless a different output folder is specified.

As further output, the DNA sequence for the primer and MSP regions can be reported, if a genome reference sequence is given as input (with parameter --reference). For the primers, the DNA sequence is reported in an additional column in the output file primerData.tsv. For the MSP regions the DNA sequences are stored in a fasta file mspRegions.fa. Additionally, annotated genes overlapping the MSP region are reported in an additional column within the MSP region output file pcrProduct.tsv, if an Ensembl (Aken *et al.* 2016) genome annotation is given by the parameter --annotation in gtf or gff file format.

### 2.2 Comparison of diffMONT with metilene and DSS on an ONT benchmarking dataset

The publicly available Oxford Nanopore Technologies (ONT) Benchmark Dataset rrms_2022.07 from ONT in November 2023 was used for comparison with metilene and DSS. The corresponding human reference genome hg38 (GCA_000001405.15) and annotation (GCA_000001405.15) were retrieved from the NCBI (Wheeler *et al.* 2007) ftp server. We calculated the retained sequencing depth per sample using mosdepth (v0.3.3) (Pedersen and Quinlan 2018).

For comparison, the ONT dataset was analyzed with metilene (v0.2–8) (Jühling *et al.* 2016), DSS (v2.44.0) (Feng *et al.* 2014) run under R (v4.2.1) (https://www.R-project.org/), and diffMONT. As input data for diffMONT, we extracted the per CpG methylation information into bedmethyl-formatted files, using the modbam2bed package (v0.9.4) (https://github.com/epi2me-labs/modbam2bed). For metilene, data was converted from bedmethyl into bedgraph file format. For better comparison, metilene parameters were adapted to require at least 10% methylation difference with --minMethDiff 10. The output of metilene was filtered with metilene_output.pl -p 0.05 -c 3 -l 60 for an adjusted *P*-value below 0.05, a minimum of 3 CpGs and a minimum length of 60 nt, respectively. Afterwards, all DMRs with mean methylation above 10% in the control group were removed. DSS was run with default parameters, requiring a minimum length of 50 nt, a minimum number of three CpG sites per DMR, and 50% of CpG sites in the DMR having *P*-values below $10^{-5}$. The DSS results were filtered to exclude DMR with mean methylation levels exceeding 10% in the control group, and keeping only DMRs with a minimum length of 60 nt. For diffMONT, default parameters were used. For runtime comparison of diffMONT with metilene and DSS, all tools were run on a 64 core processor with CPUs of model AMD Opteron(tm) Processor 6376. All tools were run without multiprocessing for better comparison, if not stated otherwise.

The overlap of predicted regions on chromosome 17 reported by diffMONT, metilene and DSS was determined with the intersect method from bedtools (v2.31.1) (Quinlan and Hall 2010) with the parameter -u and calculating the number of resulting entries.

Read methylation was visualized using the Integrative Genomics Viewer (version snapshot 05/04/2023) (Robinson *et al.* 2011). CpG island annotations for the human genome hg38 were downloaded from the UCSC genome browser (https://genome.ucsc.edu/cgi-bin/hgTables, accessed in June 2021). All plots shown were created using custom-made scripts run under R (v4.2.1) using the packages ggplot2 (v3.5.1) (Wickham 2016), ggvenn (v0.1.10) (https://CRAN.R-project.org/package=ggvenn), and dplyr (1.1.4) (https://CRAN.R-project.org/package=dplyr) unless described otherwise.

## 3 Results

In this study, we demonstrate the functionality of diffMONT, a tool designed to predict methylation-specific PCR biomarkers from nanopore sequenced DNA data, using a publicly available ONT dataset. We describe the identification of CpGs of interest, followed by the definition of potential primers and the resulting MSP regions. To validate the effectiveness of our approach, we benchmarked diffMONT against other tools with similar functionality, namely metilene and DSS.

### 3.1 Non-uniform distribution of CpGs of interest

In order to identify biomarker candidates for clinical diagnostics, we first determined the CpG sites of interest according to Fig. 2A–G. These have to be nearly unmethylated in control samples (less than 10% methylation) and show significant hypermethylation in disease samples. For the analyzed dataset, 916 924 CpGs (3,17% of CpGs in the reference genome) of the complete human genome are predicted to be differentially methylated, see Fig. 2, available as supplementary data at *Bioinformatics* online.

### 3.2 Methylation-specific PCR primer designed by diffMONT

**Figure 3:**
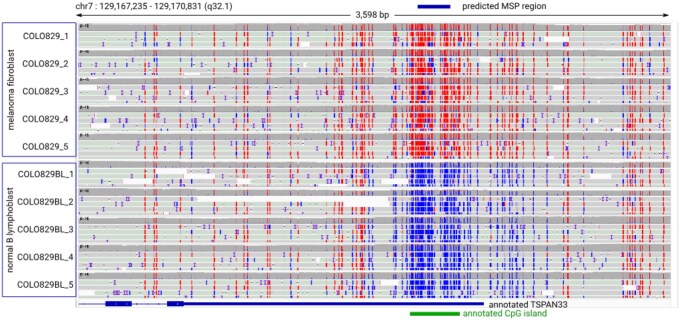
Visualization of the highest-scoring MSP region predicted by diffMONT on the ONT dataset using the Integrative Genomics Viewer (IGV). The predicted diffMONT region is annotated in blue at the top, with the gene TSPAN33 and the associated CpG island annotated below. The 5mC methylation status is color-coded: red for methylated and blue for unmethylated sites. The melanoma fibroblast sample (5×) is displayed above the normal B lymphoblast sample (5×), showing strong methylation differences overlapping the TSPAN33 3′ UTR.

For methylation-specific PCR, primers need to be 18–24 nt long, contain at least three CpGs of interest each, and receive a primer score reflecting the methylation difference between patient and control samples, see Fig. 2H and I. We provide a comprehensive list of potential primers, detailing their genomic position, length, number of CpGs of interest, score, and GC content. Applying diffMONT to the ONT dataset resulted in 9527 primer regions distributed across all human chromosomes except the Y chromosome. The majority of primers are predicted on chromosomes 1, X, 17, and 2, with 859, 767, 732, and 664 primers identified, respectively, see Fig. 3A, available as supplementary data at *Bioinformatics* online. Most potential primers of diffMONT (7050) contain the required minimum of three CpGs of interest and only fifteen primers contain seven CpGs of interest see Fig. 3B, available as supplementary data at *Bioinformatics* online. In CpG-rich regions, such as CpG islands, multiple overlapping primers are predicted (Fig. 3C, available as supplementary data at *Bioinformatics* online). As multiple combinations of predicted primers are possible to yield a PCR product (within the range of 400 nt), multiple MSP regions containing the same primers are possible (Fig. 3D, available as supplementary data at *Bioinformatics* online). The best MSP region is the combination of primers with the highest score (i.e. those primers with the highest coverage and non-control methylation).

### 3.3 MSP regions by diffMONT

**Figure 4:**
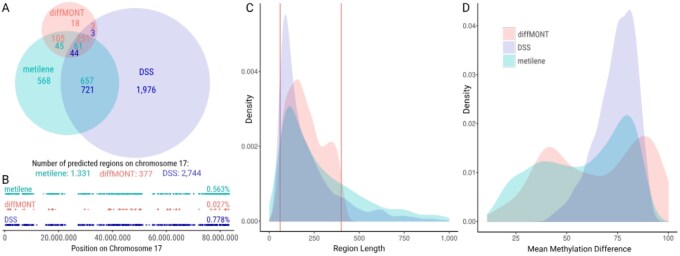
Comparison of diffMONT, metilene, and DSS on chromosome 17 using the ONT dataset. (A) Venn diagram of predicted regions found by diffMONT, metilene, and DSS. The intersections contain numbers in different colors which refer to the different tools. For example, 721 DSS regions overlap with metilene regions, whereas only 657 metilene regions overlap with DSS regions. (B) Distribution of regions predicted by diffMONT, metilene, and DSS among chromosome 17 and coverage of chromosome by the DMRs and MSP regions predicted by diffMONT, metilene, and DSS. (C) Density distribution of the length of predicted regions by diffMONT, metilene, and DSS. All regions returned by diffMONT range from 60 to 400 nt (vertical lines) due to the given values for minimum and maximum amplicon length in the algorithm (see Fig. 2J). Data is shown only for region length below 1000 nt. (D) Density distribution of the mean methylation differences between the melanoma fibroblast and normal lymphoblast replicates for diffMONT, metilene, and DSS.

The best combination of primers is selected based on the highest summed score of two primers with a user-defined distance (60–400 nt), see Fig. 2J and K. diffMONT presents a list of MSP regions containing score, coverage, and length. In total, the diffMONT algorithm reports 4872 potential MSP regions for the ONT dataset across the human genome. The highest number of MSP regions were predicted on chromosome X with 850 predicted MSP regions (see Fig. 4A, available as supplementary data at *Bioinformatics* online). The fewest hits are located on chromosomes 21, 14, and 18 with 52, 68, and 69 MSP regions, respectively. No suitable MSP region is predicted on chromosome Y, as the Y chromosome is lost in COLO829. The ONT dataset shows differences in the coverage between the chromosomes, see STab. S1, with e.g. very low coverage (0.27–0.4×) on chromosome Y for the melanoma fibroblast samples.

Most of the 4872 MSP regions overlap with annotated genomic features, as shown in Fig. 4B, available as supplementary data at *Bioinformatics* online. Specifically, 4277 MSP regions (87.77%) overlap with an annotated gene, another 210 regions (4.31%) overlap with the 500 nt upstream of a gene and about 8% (385 MSPs) are intergenic.

The score for the 4872 predicted MSP regions ranges from 415.03 to 15 042.93, see Fig. 4C, available as supplementary data at *Bioinformatics* online. The score values follow a normal distribution, with a peak at 2676. Only 57 MSP regions have a score above 10 000.

The mean methylation difference values (also called effect size), generated by subtracting the average normal B lymphoblast samples’ methylation from the average melanoma fibroblast samples’ methylation, are shown in Fig. 5A, available as supplementary data at *Bioinformatics* online reveals a high methylation difference (above 75%) for about 40% of the regions (1910 out of 4872). This is an interesting result, as the diffMONT algorithm does not require high methylation differences. In contrast, only 319 of the predicted MSP regions have a mean methylation difference below 25%.

Comparing the MSP region score with the mean methylation difference for all MSP regions predicted by diffMONT reveals a positive correlation, see Fig. 4D, available as supplementary data at *Bioinformatics* online. Regions with a higher mean methylation difference tend to have higher scores, likely because the score is influenced by the average disease methylation, and regions with significant methylation differences typically have higher disease methylation, see Fig. 5B, available as supplementary data at *Bioinformatics* online.

However, some regions exhibit a high mean methylation difference but a low score, which can be attributed to low coverage in the melanoma fibroblast samples. Since the score incorporates both disease sample methylation and coverage, low coverage results in a lower score, explaining this discrepancy.

The predicted region with the highest score overlaps an annotated CpG island as well as the 3′ UTR of the gene TSPAN33 (Tetraspanin 33), see Fig. 3, a gene for which differential methylation is linked to several lymphomas (Hevezi *et al.* 2013). The methylation for the predicted region in the TSPAN33 3′ UTR strongly differs between the melanoma fibroblast and normal B lymphoblast replicates.

Another region, which is among the 50 highest-scored MSP regions overlaps the 5′ UTR of transcription factor TCF20, see Fig. 5C, available as supplementary data at *Bioinformatics* online and a predicted CpG island. Variants of TCF20 are known to impair neurodevelopmental disability (Torti *et al.* 2019). Additionally, TCF20 is known to be abundantly expressed in small cell lung cancer (Taniwaki *et al.* 2006) and to distinguish desmoid tumors from nodular fasciitis (Bacac *et al.* 2006). The predicted MSP region shows a higher amount of CpG dinucleotides than the surrounding genomic region and a high methylation difference between the fibroblast and lymphoblast samples. The top 10 ranked MSP regions can be found in STab. S2.

### 3.4 Performance benchmark of diffMONT

To the best of our knowledge, none of the existing tools predicts MSP regions for PCRs in clinical applications. Instead, all currently available tools screen for differentially methylated regions (DMRs) in general. That is why we compared the MSP regions of our tool diffMONT with DMRs predicted by metilene and DSS. We expect our MSP regions to form a subset of DMR regions, due to our stringent selections based on (i) an applicability in a clinical context, i.e. a high methylation in disease samples and almost no methylation in control samples, instead of finding general differences in methylation; and (ii) a high methylation rate within only 24 nt allowing primer design for methylation-specific PCR.

Clinical nanopore sequencing data often has limited coverage (∼7× per replicate per flow cell for the dataset used). We focus our benchmarking on metilene, which performs well with such data (Jühling *et al.* 2016), and DSS, which is currently recommended by ONT for the analysis of nanopore sequencing data.

For comparison diffMONT, metilene, and DSS were applied to chromosome 17 of the ONT dataset. In terms of run time diffMONT, though with a shorter pre-processing time, performs much slower than metilene, but significantly faster than DSS (DSS exceeds the runtime of diffMONT by factor >80), see STab. S3.

In total, diffMONT predicted 377 MSP regions, while metilene and DSS predict 1331 and 2744 DMRs, respectively, see STab. S3. As expected, metilene and DSS predict more regions, here more than three times the number of regions predicted by diffMONT. The majority of diffMONT regions (251 of 377, 67%) were found by both tools, another 105 (28%) by metilene but not DSS, and only 3 regions by DSS but not metilene, see Fig. 4A. A high number of congruent regions (ca. 700) is detected by metilene and DSS, but not with diffMONT. This is inherent to diffMONT, being designed to detect strongly hypermethylated regions only, which additionally require almost no methylation in control samples for the following application of MSP. The coverage of the chromosome for DMR and MSP regions is for diffMONT 0.027%, for metilene 0.563%, and for DSS 0.778%, see Fig. 4B. The DMRs predicted by DSS have a required minimum length of 60 nt, and no maximum length, resulting in DMRs ranging from 60 nt to 3331 nt with a median region length of 152 nt. Nevertheless, DSS yields a high number (810) of long DMRs exceeding 250 nt. The region length distribution of DSS is similar to the one for regions predicted by metilene, with DMRs in the range of 60 nt to 3576 nt and a median region length of 228 nt, Fig. 4C. However, large regions make the PCR primer design extremely difficult, as the resulting PCR amplicon should not exceed 400 nt to ensure high enough PCR efficiency and sensitivity. Thus finding optimal primer positions for a long predicted DMR can be challenging. Regions predicted by diffMONT on the other hand have a minimum and maximum length defined (60 nt and 400 nt, respectively) and have a median region length of 189 nt. Expecting hypermethylation in the disease samples, diffMONT screens exclusively for MSP regions with a positive mean methylation difference, Fig. 4D. For metilene and DSS, the density peak of the mean methylation difference is at around 79% (metilene: 78.2%, DSS: 79.6%). This is slightly shifted compared to diffMONT (86.7%). Additionally, we reran our analyses for a subset of metilene and DSS restricting the maximum region length to 400 nt, see Fig. 6, available as supplementary data at *Bioinformatics* online. For this altered comparison, the overlap between the three tools has decreased markedly, especially the number of regions predicted by diffMONT overlapping both metilene and DSS decreased from 251 to 27 regions (Fig. 6A, available as supplementary data at *Bioinformatics* online). The other results (Fig. 6B–D, available as supplementary data at *Bioinformatics* online) remain similar. It can be summarized, that both DSS and metilene predict much more and longer regions on chromosome 17, compared to diffMONT, however, for the application of MSP in the clinical context, diffMONT seems more suitable.

## 4 Conclusion and outlook

Here we presented diffMONT, a tool to predict methylation-specific PCR biomarker regions, based on nanopore methylation sequencing data. diffMONT is easily executable via the command line. We demonstrated that the MSP regions predicted by diffMONT form a subset of the DMRs identified by other differential methylation calling tools, such as DSS and metilene. However, this subset has the advantage of showing only distinctly hypermethylated regions compared to the control sample, which is restricted to be nearly unmethylated. While diffMONT is slower than metilene, its unique advantage lies in its ability to directly generate regions suitable for primer design in MSP. This feature makes diffMONT particularly valuable for clinical applications, such as identifying biomarkers for cancer detection and screening tests. Designed with a focus on this clinical use, diffMONT simplifies and accelerates the development of methylation-specific diagnostic tests for cancer. By combining ease of use with direct applicability, diffMONT provides a practical and efficient tool for bridging the gap between epigenomic research and clinical diagnostics. The dataset used in this manuscript has some drawbacks, as it consists of one replicate only and compares cancerous fibroblasts to normal B lymphoblast cell lines instead of real patient data. However, the successful application of diffMONT on clinical data has been shown in a study (Meyer *et al.* 2025), which describes the complete workflow from nanopore sequencing of patient tissue samples, MSP region prediction with diffMONT, primer and probe design based on the predicted regions and finally validation with MSP on a larger cohort.

Currently, diffMONT does not incorporate allele-specific data (Akbari *et al.* 2021) or information regarding the primer annealing temperature. However, this design choice allows users the flexibility to apply their own tools and methods for selecting primers, depending on their specific needs and preferences. Additionally, while diffMONT is efficient in identifying MSP regions, its runtime could be further optimized by combining it with other DMR calling tools, such as metilene. Given that metilene includes nearly all regions predicted by diffMONT, an effective strategy would be to use metilene for a pre-selection step followed by diffMONT for analysis of the pre-selected regions. This combination could significantly reduce runtime while maintaining the accuracy and specificity of the MSP regions identified. These improvements would further strengthen diffMONT’s utility in clinical and research applications.

Overall, diffMONT presents a unique solution for the clinical use-case of predicting MSP-specific primer regions and thus can help to transfer knowledge of methylation-specific (cancer) markers into clinical diagnostics.

## Supplementary Material

btag039_Supplementary_Data

## Data Availability

The code of diffMONT is available at GitHub (https://github.com/rnajena/diffMONT/). Data used for the comparison as well as an archived version of the GitHub repository is available under https://zenodo.org/records/17641031.
