# Supplementary material for: diffMONT: predicting methylation-specific PCR biomarkers based on nanopore sequencing data for clinical application
Source: Bioinformatics. 2026 Jan 22;42(2):btag039. doi: 10.1093/bioinformatics/btag039 (PMC12881825; doi:10.1093/bioinformatics/btag039)
Supplement: btag039_Supplementary_Data [file btag039_supplementary_data.zip › 30-Jan-2026_024740_diffMONT_supplement.pdf]

Supplemental Figures and Tables

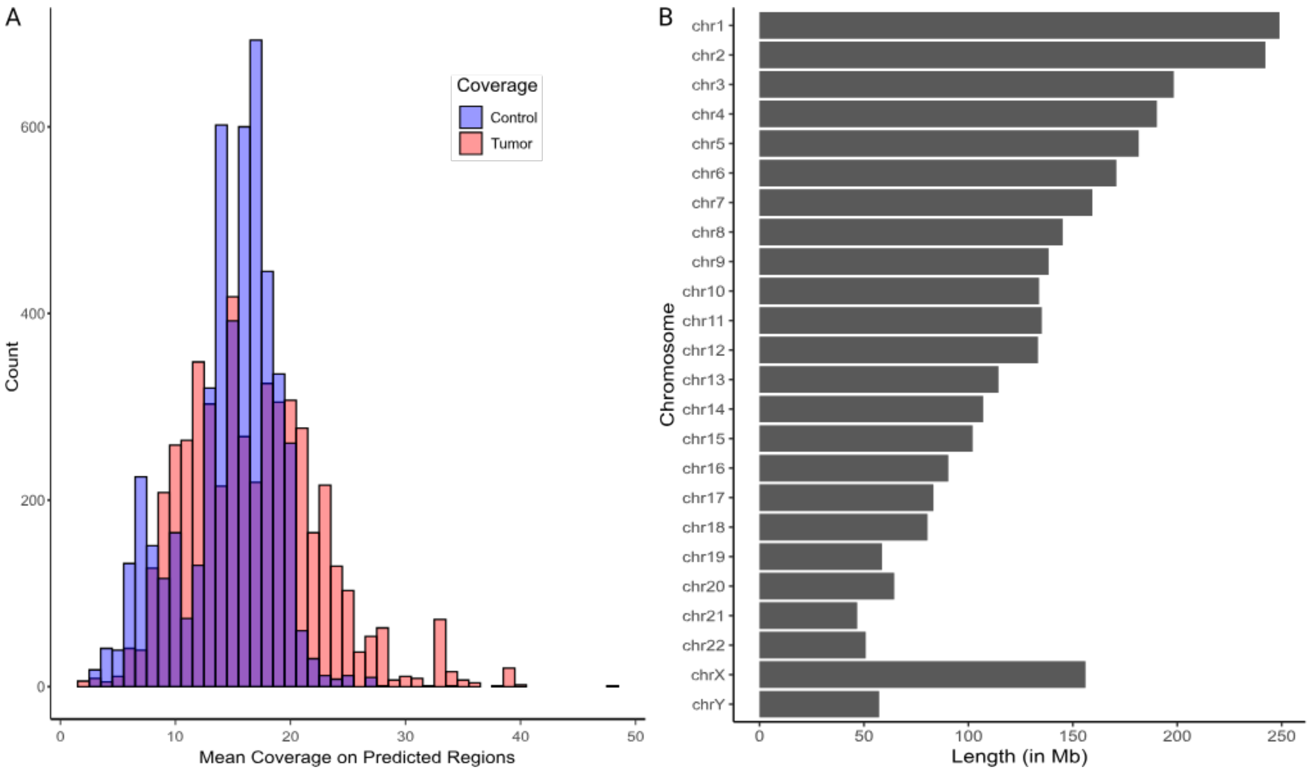

Fig. S1. (A) Histogram of the coverage distribution for all regions predicted by `diffMONT` on the ONT dataset. Data was binned for coverage, mean coverage per region of control samples and tumor samples is shown in blue and red, respectively. (B) Barplot showing the size of the different chromosomes, based on the hg38 human reference genome. Chromosome length decreases from chromosome 1 to chromosome 22. The size of chromosome X is increased compared to chromosome Y.

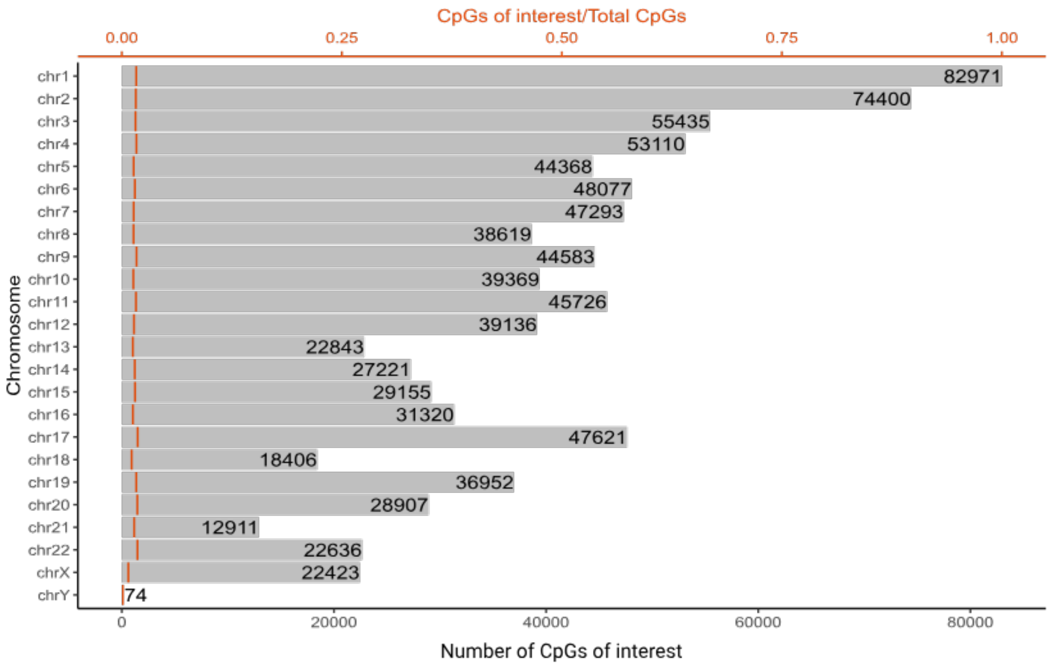

Fig. S2. Distribution of CpGs of interest across human chromosomes. As expected, the distribution largely correlates with chromosome length; however, chromosome 17 contains more CpGs of interest than anticipated. The relative amount of CpGs of interest compared to the total number of CpGs per chromosome vary less, with low fractions on chromosomes Y, X and 18.

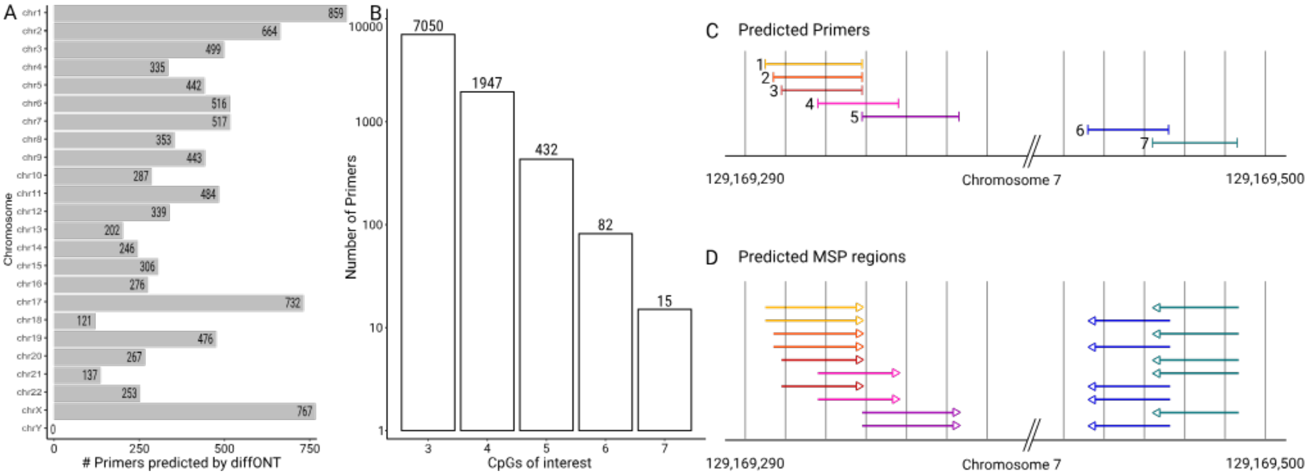

Fig. S3. (A) Distribution of primers predicted by `diffMONT` across chromosomes. The distribution generally follows chromosome length (SFig. S1 B), with exceptions on chromosomes 17 and X, which have more CpGs of interest than expected. (B) In total, 9 526 primers contain at least three CpGs of interest, with the majority of primers predicted by `diffMONT` containing exactly three CpGs of interest. Note: the y-axis is visualized on a logarithmic scale (log10). (C) Most individual primers predicted by `diffMONT` show overlap. (D) The various primer combinations generate multiple MSP regions with slightly different scores and characteristics, such as length and number of CpGs, as shown in Fig. 2K.

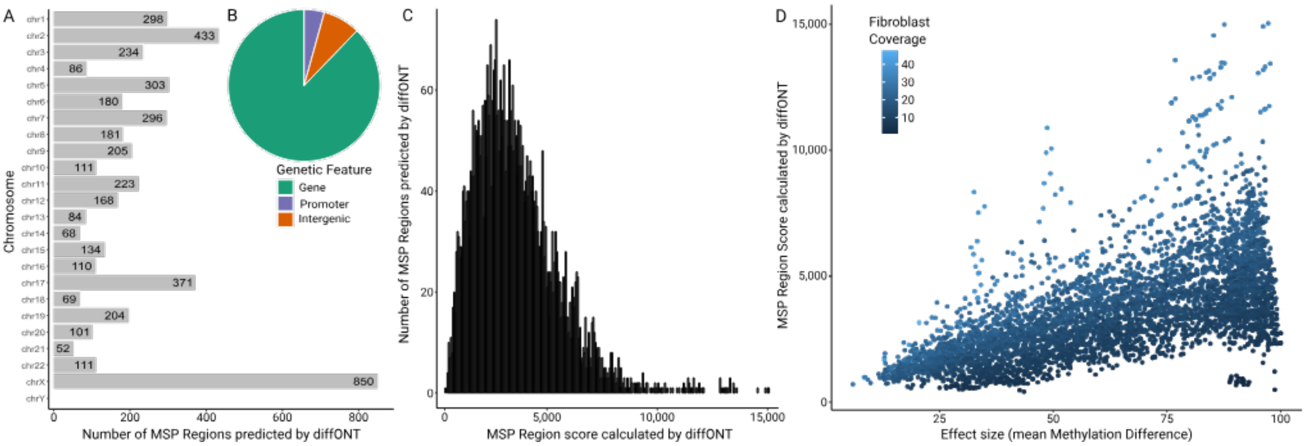

Fig. S4. (A) Distribution of predicted MSP regions by `diffMONT` on the ONT dataset across chromosomes. The regions are unevenly distributed, with the highest concentrations on chromosomes X, 2, and 17. (B) MSP regions overlap with (annotated) genomic features: 4 277 genes, 210 promoter regions (-500 nt), and 385 intergeneic regions. (C) Histogram of `diffMONT` score values for all MSP regions predicted on the ONT dataset, with scores binned in intervals of 50. The distribution peaks around 2 700, with scores above 10 000 appearing as outliers. (D) Visualization of MSP region scores versus mean methylation differences between melanoma fibroblast and normal B lymphoblast samples for all MSP regions predicted by `diffMONT` on the ONT dataset. Color represents average coverage in melanoma fibroblast samples. Regions with low scores despite high methylation differences (bottom right) exhibit low coverage in melanoma fibroblast samples.

Table S1. Sequencing coverage per chromosome per flowcell, calculated with mosdepth Pedersen and Quinlan [2018]. Sequencing coverage shows differences between the chromosomes over all samples. Especially chromosome Y but also chromosome X show a low coverage (Coverage below 1X for all COLO829 flowcells and below 2X for all COLO829BL samples for chromosome Y). On the other hand, chromosomes 19 and 22 show the highest overall coverage with a mean coverage of 16.76 X on chromosome 19 and 12.89 X on chromosome 22.

Chr	COLO829					COLO829BL				
	FC 1	FC 2	FC 3	FC 4	FC 5	FC 1	FC 2	FC 3	FC 4	FC 5
chr1	8.07	7.81	5.80	7.51	7.86	8.39	6.96	7.32	7.13	7.74
chr2	7.29	7.04	5.31	6.76	7.12	7.11	5.95	6.20	6.06	6.58
chr3	8.59	8.26	6.26	7.95	8.37	6.76	5.68	5.88	5.76	6.26
chr4	7.13	6.82	5.19	6.54	6.95	6.27	5.30	5.47	5.33	5.80
chr5	4.55	4.37	3.31	4.18	4.44	6.62	5.55	5.76	5.65	6.12
chr6	6.63	6.43	4.81	6.05	6.5	6.96	5.86	6.05	5.92	6.40
chr7	11.59	11.16	8.35	10.72	11.21	8.30	6.93	7.25	7.06	7.65
chr8	7.30	7.05	5.32	6.75	7.12	7.04	5.92	6.12	5.98	6.49
chr9	9.12	8.80	6.56	8.42	8.86	7.38	6.16	6.45	6.30	6.80
chr10	5.86	5.64	4.20	5.39	5.65	8.29	6.92	7.24	7.06	7.66
chr11	7.96	7.69	5.74	7.42	7.73	8.31	6.9	7.21	7.02	7.64
chr12	8.51	8.18	6.15	7.91	8.21	8.28	6.88	7.21	7.03	7.64
chr13	5.85	5.62	4.27	5.36	5.73	5.44	4.6	4.78	4.64	5.05
chr14	6.80	6.57	4.92	6.33	6.64	6.68	5.57	5.84	5.70	6.19
chr15	5.81	5.62	4.21	5.42	5.65	6.69	5.52	5.82	5.68	6.14
chr16	9.62	9.20	6.82	8.94	9.26	11.06	9.14	9.69	9.44	10.21
chr17	14.08	13.59	10.05	13.3	13.53	13.52	11.05	11.78	11.43	12.37
chr18	4.62	4.48	3.35	4.26	4.55	6.57	5.54	5.74	5.61	6.10
chr19	18.96	18.22	13.45	17.84	18.17	18.19	14.8	15.84	15.40	16.73
chr20	14.22	13.75	10.21	13.28	13.68	10.33	8.53	9.03	8.81	9.50
chr21	11.12	10.65	7.85	10.14	10.60	10.08	8.42	8.89	8.59	9.32
chr22	16.53	15.91	11.69	15.51	15.83	12.01	9.84	10.44	10.12	10.97
chrX	4.28	4.11	3.11	3.93	4.17	3.20	2.70	2.79	2.74	2.97
chrY	0.40	0.38	0.27	0.34	0.38	1.99	1.69	1.75	1.69	1.85

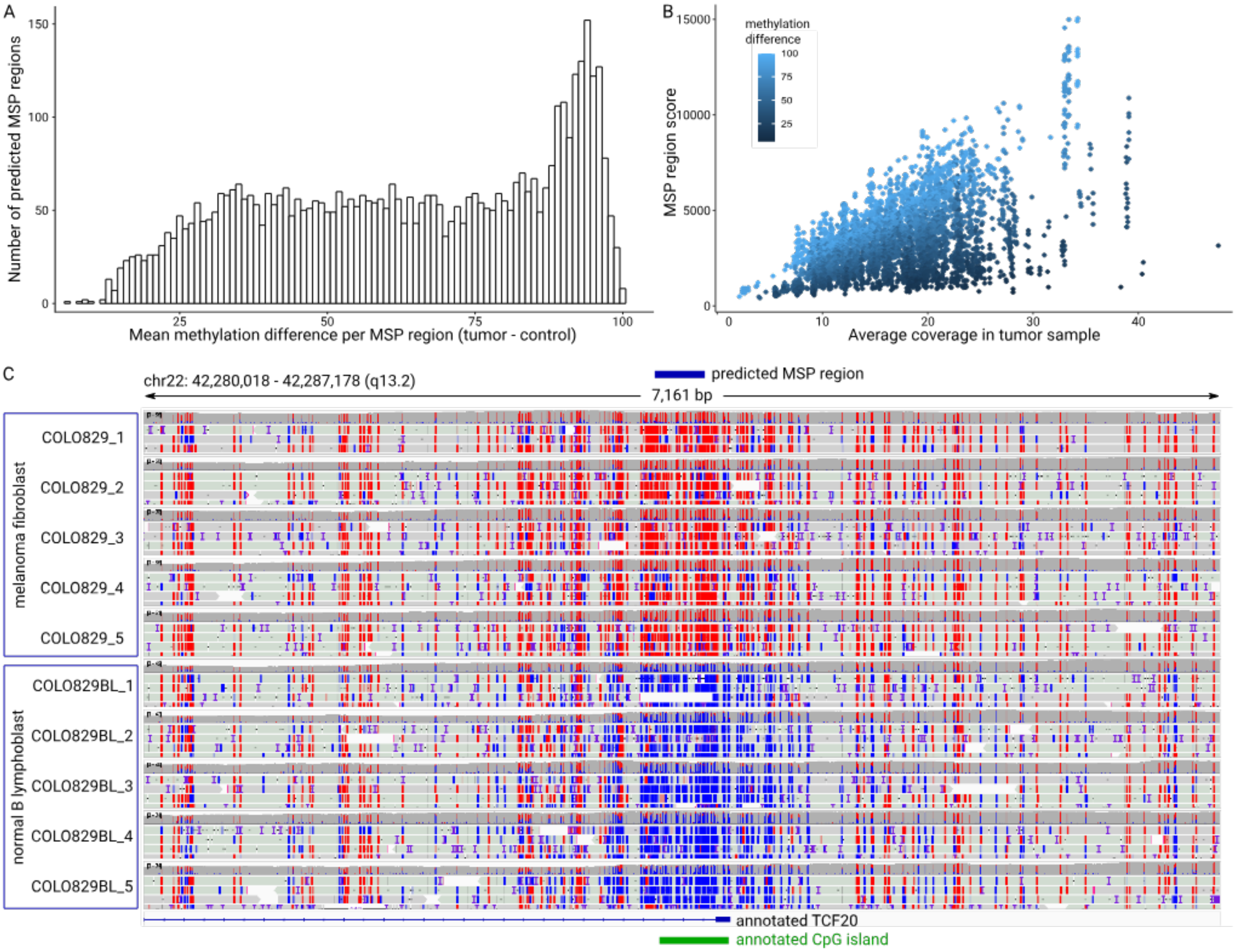

Fig. S5. (A) Histogram of the methylation difference values of the regions predicted by `diffMONT` on the ONT dataset. The methylation difference is calculated by subtracting the average melanoma fibroblast samples' methylation from the average normal B lymphoblast samples' methylation. (B) Visualization of the MSP region score and average methylation in tumor samples for all MSP regions, predicted by `diffMONT` on the ONT dataset. Only few outlier samples show a coverage below 8 X for the diseased samples. (C) Visualization of one of the top 50 reported MSP regions predicted by `diffMONT` on the ONT dataset using the Integrative Genomics Viewer (IGV). At the top the region predicted by `diffMONT` is annotated in blue; at the bottom the gene TCF20 is annotated in blue, and an annotated CpG island in green. The 5mC methylation status is color-coded, red = methylated, blue = unmethylated. The melanoma fibroblast sample is shown on top (5x), the normal B lymphoblast sample at the bottom (5x). The predicted MSP region shows strong methylation differences overlapping the TCF20 5' UTR and promoter region and CpG island.

Table S2. Result statistics for the ten highest scored MSP regions predicted by `diffMONT`.

chrom.	strand	start_fw	end_rev	length	score	cov contr	cov tumor	meth contr	meth tumor
chr7	+	129169295	129169493	198	15042.93	15.18	34.24	0.30	97.57
chr7	-	128031667	128031777	110	14991.92	12.95	33.40	0.00	87.62
chr7	+	129169295	129169476	181	14917.33	15.15	34.20	0.30	96.46
chr7	-	128031397	128031690	293	14555.40	13.45	33.00	0.00	85.26
chr7	-	128031667	128031740	73	13581.43	13.00	33.40	0.26	77.07
chr7	-	128031623	128031690	67	13480.56	13.02	33.30	0.00	87.00
chr7	-	128031669	128031777	108	13456.56	12.90	33.40	0.00	87.68
chr7	+	129169297	129169493	196	13438.65	15.18	34.24	0.20	97.90
chr7	+	129169297	129169476	179	13313.05	15.15	34.20	0.20	96.78
chr7	-	128031671	128031777	106	13309.22	12.90	33.40	0.00	86.86

Table S3. Comparison of the main result statistics between `diffMONT`, `metilene`, and `DSS` on the ONT benchmarking dataset. Time calculated for pre-processing starting with (unsorted) bedmethyl files. # regions – number of DMRs and MSP regions, respectively.

	diffMONT	metilene	DSS
pre-processing time	33 min.	97 min.	20 min.
runtime (chr17)	28 min.	3 min.	2 372 min.
# regions (chr17)	377	1 331	2 744

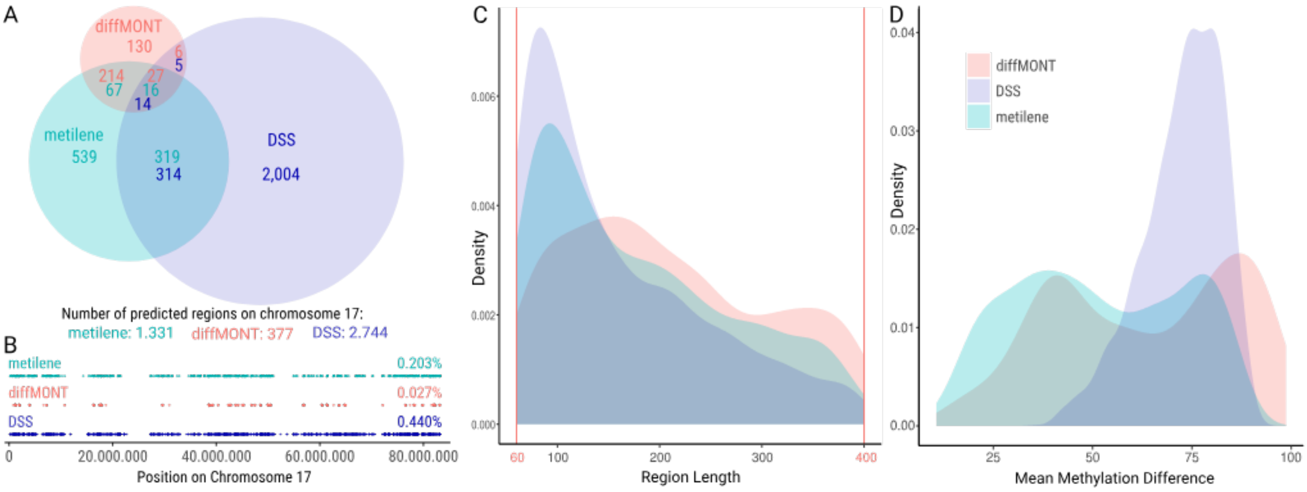

Fig. S6. Comparison of `diffMONT`, `metilene`, and `DSS` on chromosome 17 using the ONT dataset containing olny regions with a length of 400 nt at most. (A) Venn diagram of predicted regions found by `diffMONT`, `metilene`, and `DSS`. The intersections are altered compared to Fig. 4 by generally lower overlaps between the regions predicted by the three tools. (B) Distribution of regions predicted by `diffMONT`, `metilene`, and `DSS` among chromosome 17 and coverage of chromosome by the DMRs and MSP regions predicted by `diffMONT`, `metilene`, and `DSS` is decreased for `metilene` and `DSS` compared to Fig. 4. (C) Density distribution of the length of predicted regions by `diffMONT`, `metilene`, and `DSS`. (D) Density distribution of the mean methylation differences between the melanoma fibroblast and normal lymphoblast replicates for `diffMONT`, `metilene`, and `DSS`. The general distribution is unchanged compared to Fig. 4, though for `metilene` the peak at 75 % is decreased in height.

Supplemental Results

Whole genome comparison with metilene

metilene was implemented to study DNA methylation differences, e.g., after environmental stress in plants López et al. [2022], during development Gilsbach et al. [2018], Hamazaki et al. [2021], and for the prediction of cancerous lung tissue Cui et al. [2024]. We applied metilene to predict DMRs to the same ONT dataset analyzed with diffMONT. In total, metilene predicted 16 643 DMRs, of which only 1 157 overlap with MSP regions of diffMONT, see Fig. S7A. Contrarily, 4 401 (90 %) of 4 872 MSP regions predicted by diffMONT overlap with a DMR predicted by metilene. There are mainly two differences between the regions predicted by diffMONT and metilene: (i) the length of metilene's DMRs ranges from 60 nt to 4 163 nt, whereas diffMONT's MSP regions range from 60 nt to 400 nt, see Fig. S7B, resulting in generally longer regions predicted by metilene, additionally, (ii) diffMONT has specific requirements for the methylation pattern, whereas metilene predicts hyper- and hypomethylated regions more generally. These two differences explain the fraction of metilene DMRs not intersecting with MSP regions. The density distribution of mean methylation differences of the regions predicted by metilene ranges from 0 to 100 with a peak 80 % methylation difference. For the regions predicted by diffMONT, the density peak of the methylation difference is at 92, thus diffMONT focuses on regions with slightly higher methylation differences, see Fig. S7C, green. Notably, for all the comparisons made, the diffMONT score filter has not been applied at this stage, thus, applying it will further increase the rate of methylation differences among the finally selected MSP regions, leading to even more accurate results of diffMONT. In order to understand the distribution of methylation differences in more detail, we further distinguished the four fields of the Venn Diagram from Fig. S7A in Fig. S7D. The methylation difference of the predicted subgroups follow similar distributions.

The distribution, number, and appearance of diffMONT and metilene regions across the chromosomes are reported in Tab. S4, Fig. S7E (note the log-scaled x-axis) and Fig. S7F. Both tools predict no methylation differences on the 5' ends of acrocentric chromosomes 13, 14, 15, 21, and 22.

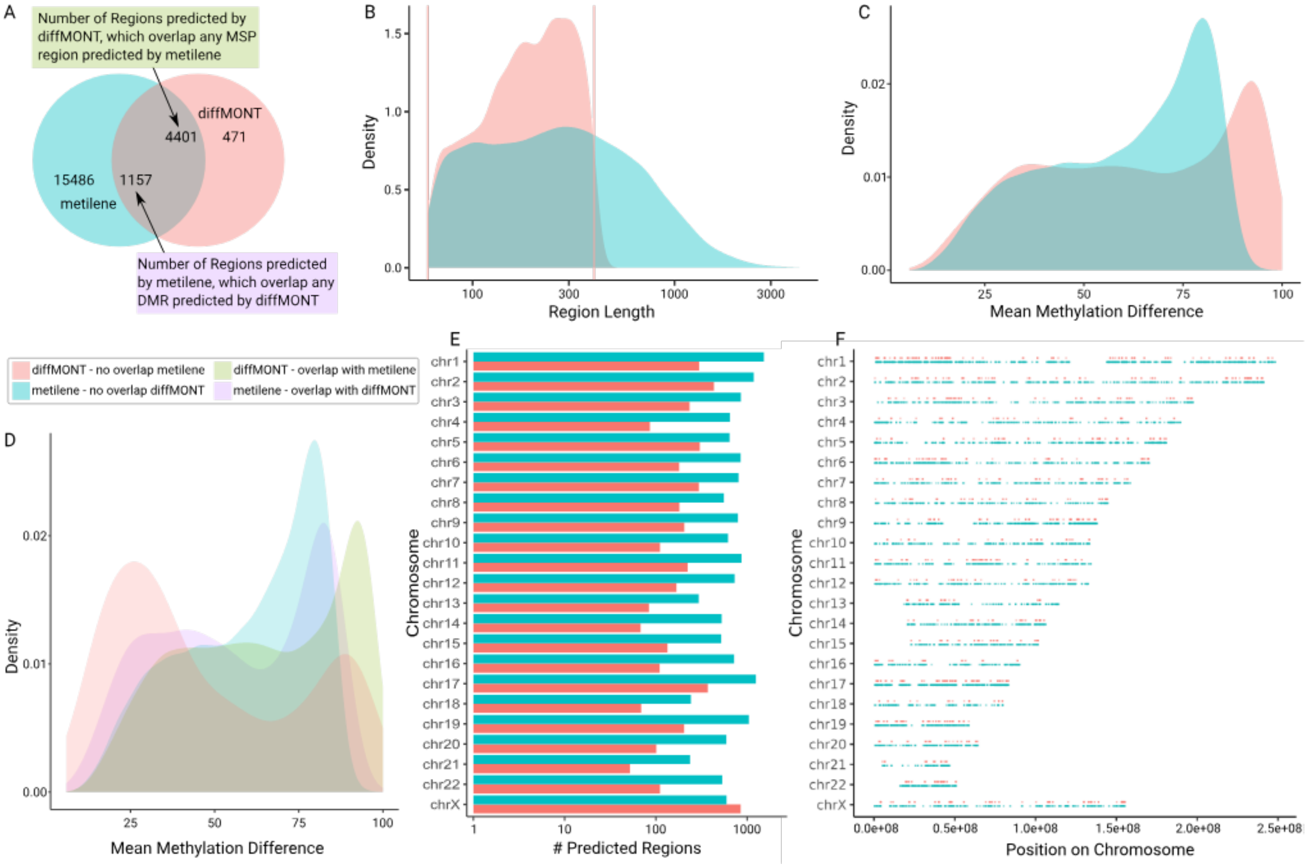

Fig. S7. Comparison between diffMONT (red) and metilene (green) for the ONT dataset, comparing cancerous fibroblast cells with normal lymphoblast cells. (A) Venn diagram showing the overlap of predicted regions. (B) Frequency density of the region length. All regions returned by diffMONT range from 60 to 400 nt (vertical lines) due to the given values for minimum and maximum amplicon length in the algorithm, see Fig. 2J. Please note: x-axis is shown in log(10) scale. (C) Frequency density of the mean methylation differences between the melanoma fibroblast and normal lymphoblast replicates. (D) The distribution of the four fields of the Venn diagram. (E) Number and (F) distribution of DMR (from metilene) and MSP (from diffMONT) regions per chromosomes.

In terms of run time diffMONT, though with a shorter pre-processing time, performs much slower than metilene. However, diffMONT can predict highly methylated regions more precisely, see Tab. S3. diffMONT is computationally even more intensive for

Table S4. Number of regions called per chromosome by diffMONT and metilene on the ONT dataset. For both methods the distribution of predicted regions among the chromosomes is quite similar among the chromosomes. While diffMONT predicts most regions on chromosome X, metilene predicts most regions on the chromosomes 1, 17, 2, and 19. Both methods predict the least regions on chromosome 21. Low counts also exist on alternative chromosomes (data not shown). Interestingly, running metilene for the whole genome resulted in a slightly differing number of predicted regions on chromosome 17 (1 248 vs. 1 331 predicted regions for chromsome 17).

chr	diffMONT	metilene
1	298	1 527
2	433	1 182
3	234	854
4	86	648
5	303	643
6	180	849
7	296	806
8	181	556
9	205	793
10	111	618
11	223	868
12	168	730

chr	diffMONT	metilene	DSS
13	84	295	
14	68	526	
15	134	520	
16	110	720	
17	371	1 248	2 744
18	69	242	
19	204	1 046	
20	101	592	
21	52	237	
22	111	534	
X	850	595	
Y	0	0	

Table S5. Comparison of the main result statistics between diffMONT and metilene on the ONT benchmarking dataset. Time calculated for pre-processing starting with (unsorted) bedmethyl files. # regions – number of DMRs and MSP regions, respectively.

	diffMONT	metilene	DSS
pre-processing time	33 min.	97 min.	20 min.
runtime (genome)	570 min.	50 min.	-
# regions (genome)	4 872	16 643	-

whole-genome analysis and more efficient for smaller regions, such as individual chromosomes. The longer runtime is initially counterintuitive given the smaller number of regions diffMONT predicts. It arises, however, due to single nucleotide analysis and calculation of single nucleotide statistics (Fig. 2E).

However, we can assume diffMONT to predict hypermethylated regions more precisely than metilene, with higher accuracy.

Comparison with pycoMeth

Additionally, we show benchmarking data for pycoMeth, as this tool has been developed especially for DMR detection in nanopore sequencing data.

pycoMeth Snajder et al. [2023] is to our knowledge the only tool designed for differential methylation analysis using nanopore sequencing data processed through Nanopolish. pycoMeth shows increased recall and precision in DMR testing, especially for detecting low effect-size methylation changes in low-coverage settings Snajder et al. [2023]. Additionally, by utilizing Bayesian changepoint detection, pycoMeth accommodates methylation call uncertainties instead of binarization of methylation probabilities as the other tools described above Snajder et al. [2023]. pycoMeth is suitable for comparing two or more samples. pycoMeth focuses on identifying methylated regions based on nanopore sequencing data. However, it fails to support newer methylation callers such as implemented in the current state-of-the-art basecaller Dorado[6].

GIAB benchmarking dataset for comparison with pycoMeth

We could not use the same benchmarking dataset for pycoMeth as for the other tools because pycoMeth relies on methylation calls from nanopolish Simpson et al. [2017]. Re-basecalling and methylation calling with nanopolish required the original fast5 raw data, which was corrupted in the ONT benchmarking dataset, rendering it unusable.

Therefore, for the comparison of diffMONT with pycoMeth, we used the benchmarking dataset from the pycoMeth publication Snajder et al. [2023]. However, this GIAB dataset does not contain cancer-specific data. As a result, the comparison with metilene and DSS was conducted using the ONT benchmarking dataset, which includes the relevant cancer-specific data. We downloaded the samples HG003[7] and HG004[8] from the Ashkenazim Trio from the GIAB consortium Zook et al. [2016]. These samples are from a healthy human male and female, respectively. Basecalling of the fast5 raw data was performed with Guppy (v6.5.7)[9], using the model dna_r9.4.1_450bps_modbases_5mc_cg_hac.cfg; methylation calling was performed with nanopolish (v0.14.0) Simpson et al. [2017]. For MSP region prediction with diffMONT, the bam output files generated by Guppy were merged, sorted and indexed with SAMtools (v1.16.1) Danecek et al. [2021] and converted into bedmethyl file format using modbam2bed (v0.9.4)[10],

[6] https://github.com/nanoporetech/dorado

[7] https://s3-us-west-2.amazonaws.com/human-pangenomics/NHGRI_UCSC_panel/HG003/nanopore/GM24149_1.fast5.tar.gz accessed in August 2024

[8] https://s3-us-west-2.amazonaws.com/human-pangenomics/NHGRI_UCSC_panel/HG004/nanopore/GM24143_1.fast5.tar.gz accessed in August 2024

[9] accessible in the Nanopore Community https://nanoporetech.com/community.

[10] https://github.com/epi2me-labs/modbam2bed

before MSP regions were predicted using `diffMONT` with the parameters `--minCtrCov 0` and `--minCtrls 0`. For `pycoMeth` the DMR detection was performed on the GIAB dataset with default parameters, by running first `CpG_Aggregate`, then segmenting the genome into intervals based on methylation by using `Meth_Seg` and finally using `Meth_Comp` of `pycoMeth` Snajder et al. [2023] to check for differential methylation for each interval.

For runtime comparison of `diffMONT` and `pycoMeth`, both tools were run on a 64 core processor with CPUs of model AMD Opteron(tm) Processor 6376. Both tools were run with default settings for number of threads, if not stated otherwise.

pycoMeth predicts more and longer regions than diffMONT

`pycoMeth` was the first tool being developed to predict DMRs from nanopore data, which includes storage, management and analysis of ONT DNA methylation data Snajder et al. [2023]. Additionally, to our knowledge, `pycoMeth` is currently the only tool using low-coverage (up to 15 X) long-read nanopore sequencing data for predicting DMRs. For the comparison of `diffMONT` to `pycoMeth`, both tools were applied to a male and a female sample, resulting in more than 100 000 predicted regions for each of the tools, Tab. S6.

Table S6. Distribution of regions predicted by `diffMONT` and `pycoMeth` on the GIAB dataset. In total, 104 805 and 157 660 regions were identified for `diffMONT` and `pycoMeth`, respectively. No regions are predicted on chromosome Y, since female cells were compared to male cells. Low counts also exist on alternative chromosomes (data not shown).

chr	diffMONT	pycoMeth	chr	diffMONT	pycoMeth
chr1	1 235	11 117	chr13	239	5 927
chr2	530	12 153	chr14	196	5 813
chr3	509	9 662	chr15	300	4 522
chr4	331	8 944	chr16	873	5 036
chr5	577	9 383	chr17	483	5 860
chr6	435	8 783	chr18	202	4 224
chr7	199	8 814	chr19	375	5 196
chr8	571	7 441	chr20	256	3 372
chr9	251	7 523	chr21	575	2 520
chr10	359	8 641	chr22	202	2 373
chr11	266	8 190	chrX	95 527	4 346
chr12	282	7 797	chrY	–	–

`pycoMeth` segments the genome based on methylation information into intervals, resulting in 584 171 intervals for the GIAB dataset. Differential methylation analysis of these intervals identified 157,660 intervals as DMRs, characterized by significant p-values. While about half of the MSP regions (54 122 out of 104 805) predicted by `diffMONT` overlap a region predicted by `pycoMeth`, only a small fraction of regions predicted by `pycoMeth` (422 out of 157 660) are overlapping an MSP region, Fig. S8A. At first glance, this may appear to be a disproportionate distribution; however, it can be explained by the following observation: While the MSP regions predicted by `diffMONT` have a defined length of 60-400 nt, the intervals predicted by `pycoMeth` range from 0 to 2 348 466 nt in length, see Fig. S8B, with a peak at 4 596 nt. Note, that `DSS` and `metilene` have not identified any such long DMR, see Fig. S7. If many short `diffMONT` MSP regions overlap with one long `pycoMeth` DMR region, then this results in a high overlap of MSP regions with DMRs and a small number of DMRs overlapping with any MSP result. The number of MSP regions predicted by `diffMONT` for the GIAB dataset is approximately 15 times higher than the number predicted for the ONT dataset, generally indicating more methylation differences between males and females than between healthy and cancerous male cells. Interestingly, the MSP regions predicted by `diffMONT` are distributed unequally among the chromosomes. An extremely high amount of predicted regions can be seen on chromosome X (95 527, ∼91 %), with the second-highest amount of MSP regions predicted on chromosome 1 (1 235), Fig. S8C. This can be explained by the fact, that DNA methylation on the X chromosome reflects sex-specific dosage compensation driven by X-chromosome inactivation (XCI) in the female sample, which has two X chromosomes Duncan et al. [2018].

Most of the DMRs predicted by `pycoMeth` are located on chromosome 2 (12 153) and chromosome 1 (11 117) (Fig. S8C, Tab. S6). `pycoMeth` does not predict an increased amount of DMRs on chromosome X (4 346). Instead, the distribution of DMRs predicted by `pycoMeth` follows the chromosome length distribution, SFig. S1. As `diffMONT` is designed to show methylation differences detectable in an MSP, `diffMONT` screens for methylation differences in one direction only, originally higher methylation in tumor samples compared to control samples. `pycoMeth` on the other hand, is not designed for this specific approach and thus predicts both hyper- and hypomethylated regions. As Fig. S8D displays, all `diffMONT` predicted MSP regions are hypermethylated, i.e., showing higher methylation levels in the female sample. The majority of these predicted MSP regions display a methylation difference exceeding 50 %. As expected, `pycoMeth` predicts regions with both positive and negative methylation differences between the samples. However, most regions predicted by `pycoMeth` show a methylation difference below 50 % and thus are difficult to use in an MSP.

For comparison of runtime, we excluded basecalling and methylation calling. Running the `pycoMeth` workflow took 31 590 min. in total. The workflow consists of the short step `meth5 create_m5`, followed by the methylation-based segmentation of the genome `pycometh Meth_Seg`, which took 31 293 min., and is advised to be performed chromosome-wise. The final step `pycometh Meth_Comp` took 104 min. Despite a longer preprocessing time (421 min.), `diffMONT` is in total much faster than `pycoMeth` (1216 min.), see Tab. S7.

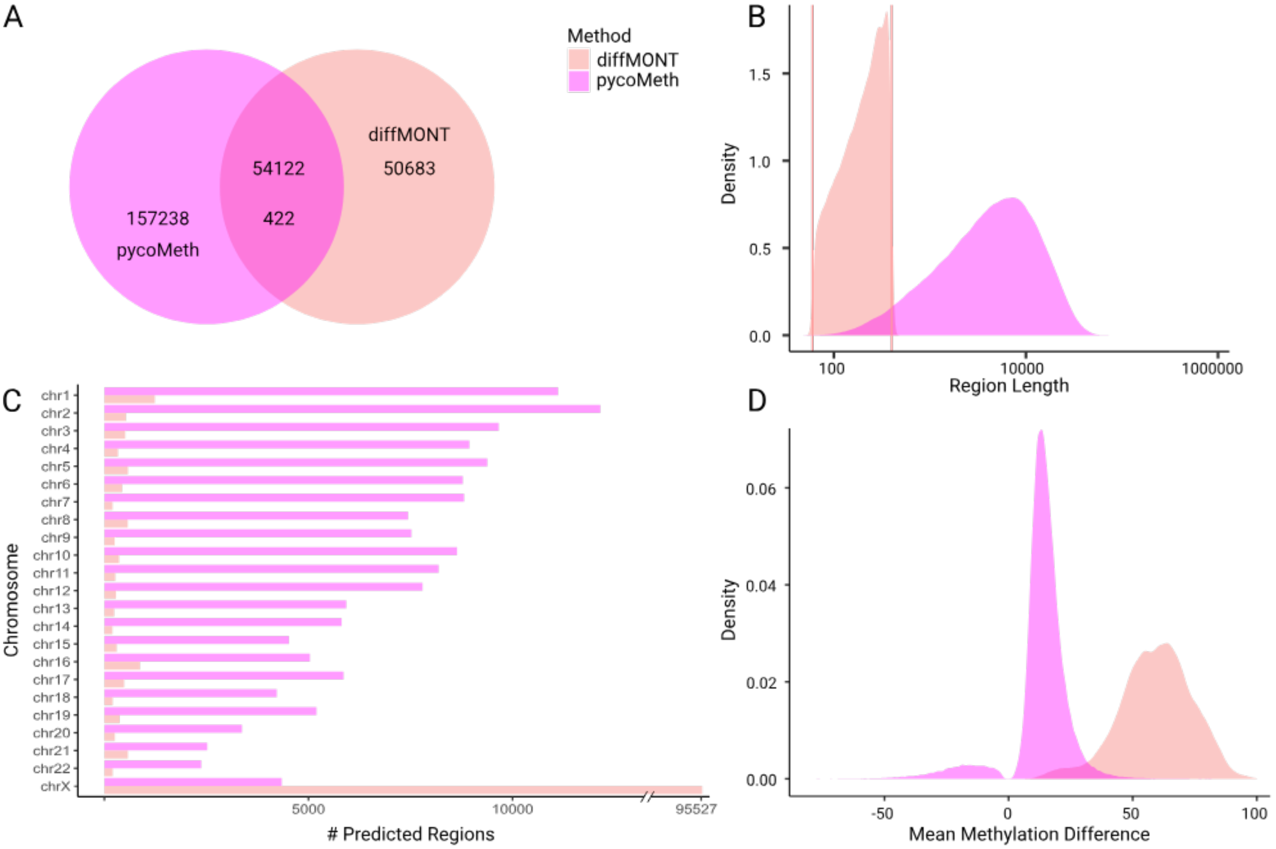

Fig. S8. Comparison between `diffMONT` (red) and `pycoMeth` (purple) for the GIAB dataset. (A) Venn diagram showing the overlap of predicted regions. (B) Density distribution of the region length of predicted regions by `diffMONT` and `pycoMeth`. All regions returned by `diffMONT` range from 60-400 nt (vertical lines) due to the given values for minimum and maximum amplicon length in the algorithm, see Fig. 2J. The X-axis is shown in log(10). The regions predicted by `pycoMeth` far exceed the length of those predicted by `diffMONT`. (C) Distribution of the `diffMONT` and `pycoMeth` predicted regions among the chromosomes. The X chromosome includes more than 95 500 (91 %) of the `diffMONT` predicted regions. (D) Density distribution of the methylation difference of predicted regions by `diffMONT` and `pycoMeth`. Methylation above 0 means higher methylation in the female sample HG004.

Table S7. Comparison of the main result statistics for `diffMONT` and `pycoMeth` on the GIAB dataset.

	`diffMONT`	`pycoMeth`
pre-processing time	421 min.	0
runtime	795 min.	31 590 min.
# predicted regions	104 773	157 637
